# Report of the Court of Examiners to the Society of Apothecaries

**Published:** 1836-10

**Authors:** 


REPORT OF THE COURT OF EXAMINERS TO THE SOCIETY OF APOTHECARIES.

THE Court of Examiners, in reporting to the Master, Wardens, and Court of Assistants, the result of their labours during the past year, have the honour to state, that the total number of candidates examined during that period amounts to 556, being considerably above the number of any former year.

Within that period, 450 gentlemen have received their certificates of qualification; of whom, several have shown so great a proficiency in every branch of their medical studies, as to have received the especial commendations of the Court, and 106 have been directed to return to their studies for the period prescribed by the act of parliament; of which number, thirty-six have been rejected solely on account of their defective knowledge of the Latin language.

The Court regret extremely that they are compelled to report so large a proportion of rejections during the last year, amounting nearly to one in every five candidates, and they cannot but lament that ignorance of the Latin language still continues to be of such frequent occurrence; and this is the more to be regretted, as the attention of the profession has been called repeatedly, during the last twenty years, to this particular point.

In order more efficiently to test the classic attainments of the candidates, and at the same time to enable them subsequently to devote their time exclusively to their medical studies, the Court instituted last year a preliminary examination in the Latin medical classics, which came into operation on the 30th May, 1835, and has been continued every succeeding Saturday, under the direction of three members of the Court, who take this duty in rotation. Since that time, 1200 students have undergone this examination. The Court have reason to know that this plan has proved highly acceptable to the students, not only in London, but throughout the whole of the provincial schools.

The Court beg to subjoin the number of the registrations in the London and provincial schools in October, 1835:—

*Of Pupils Registered.*

| | |
|---|---|
| In the provincial schools . . . . . . | 322 |
| London . . . . . . . . . . . . . . . . | 640 |
| Total . . . . . . | 962 |

J. Bacot, Chairman.

*Apothecaries' Hall, July* 28, 1836.

In reference to the last clause but one of the foregoing Report, we beg leave to subjoin a few remarks which we made when the subject first came before us, and which we reprint from the second number of our journal, p. 519. To these remarks we respectfully call the attention of the Court of Examiners.

"We have not time at present to enter upon the subject of the preliminary education requisite for the medical student, of which the study of the Latin language constitutes so important a part; but we must be permitted to observe, in passing, that, if the recent regulation adopted by the Apothecaries' Company, of admitting young men to their classical examination on *commencing* their studies in London, be designed to exclude all further testing of the candidate's proficiency in the Latin language, when they present themselves for the licence after completing their medical studies, the rule is, in our opinion, most injudicious, and calculated to perpetuate and increase the very evils it was intended to mitigate or remove. It is surely not merely desirable that the student should give evidence that he has acquired a competent knowledge of the Latin language, previously to entering on the serious business of studies more strictly professional, but also that he preserves this knowledge during the progress of these studies, and carries it into practice with him. It would, no doubt, be of advantage to a medical student to have undergone the intellectual training implied by the study of the ancient languages, even if, on entering upon his profession, he were to lose all practical knowledge of them; but surely it will be equally admitted that the preservation of the knowledge acquired in the classical school-room, must be at least of as great importance to the practitioner of medicine. Now, does not the regulation alluded to almost seem to imply—(it being assumed that this preliminary examination is the only one in classics,) that the student, having once exhibited the requisite degree of competency in Latin, may for ever after disregard it? Or, at least, will not this be, in fact, the practical operation of the regulation on the minds of a considerable number of students? The learned directors of the Apothecaries' Company may have had a truer and more disinterested love of Latin for itself, and a more astringent memory for vocables and syntax, than we could ever boast of; but, for our own parts, we are bound, by the faith of honest criticism, although with shame and confusion of face, to acknowledge that, had it not been for the consciousness ever present to us during our medical studies, that we should have to *make our appearance in Latin* at the end of them, we might, in our free zeal for *science*, have actually forgot, if not all, at least much of the *literature* that had been forced upon us in our earlier years. We shall heartily rejoice to find that there is a difference in this respect between the students of present and former times, and that the good intentions of the Apothecaries' Company, which we do not for one moment call in question, will be fulfilled, and our fears proved to be without foundation: we, however, to say the least, entertain very strong doubts on the subject." (*British and Foreign Med. Review*, No. II. p. 519.)

